# Correlation of Apiose Levels and Growth Rates in Duckweeds

**DOI:** 10.3389/fchem.2018.00291

**Published:** 2018-07-20

**Authors:** Débora Pagliuso, Adriana Grandis, Eglee S. Igarashi, Eric Lam, Marcos S. Buckeridge

**Affiliations:** ^1^Laboratory of Plant Physiological Ecology, Department of Botany, Systems and Synthetic Biology Center, Institute of Biosciences, University of São Paulo, São Paulo, Brazil; ^2^Department of Plant Biology, Rutgers, The State University of New Jersey, New Brunswick, NJ, United States

**Keywords:** duckweed, lemnoideae, wolffioideae, starch, cell wall, apiose, growth

## Abstract

The carbon assimilated by photosynthesis in plants can be partitioned into starch, soluble sugars, and cell wall polymers. Higher levels of starch accumulation in leaves are usually correlated with a lower growth capacity. Duckweeds are fast-growing aquatic monocot plants that can accumulate high levels of starch. They are an unusual group because their cell wall has very low levels of lignin while accumulating apiogalacturonan, a pectic polysaccharide that could be involved with boron assimilation. In this work, five duckweed species from different genera (*Spirodela polyrhiza, Landoltia punctata, Lemna gibba, Wolffiella caudata*, and *Wolffia borealis*) were cultivated under two light intensities (20 and 500 μmoles of photons m^−2^ s^−1^) to evaluate the effects of growth rate on carbohydrate metabolism. A comparative analysis was performed by measuring their relative growth rates (RGR), and their content for starch, as well as soluble and cell wall carbohydrates. We found that the faster-growing species (the Lemnoideae) accumulate lower starch and higher soluble sugars than the slower-growing species within the Wolffioideae. Interestingly, analysis of the cell wall monosaccharides revealed that the slower-growing species displayed lower content of apiose in their walls. Our results indicate that higher accumulation of apiose observed in cell walls of the Lemnoideae species, which likely correlates with a higher proportion of apiogalacturonan, may lead to higher efficiency in the assimilation of boron. This is consistent with the increased RGR observed under conditions with higher apiose in the cell wall, such as higher light intensity. Consistent with their lower growth capacity, the Wolffioideae species we studied shows higher starch accumulation in comparison with the Lemnoideae species. We suggest that apiose levels could be good biomarkers for growth capacity of duckweeds and suggest that boron uptake could be an important factor for growth control in this aquatic plant family.

## Introduction

Duckweeds (the family Lemnaceae) are the smallest monocots and live as free-floating aquatic plants (Landolt, 1992; Appenroth et al., 2013). The 37 species of Lemnaceae have been classified into five genera (*Spirodela, Landoltia, Lemna, Wolffiella*, and *Wolffia*) based on their morphology and physiology (Borisjuk et al., 2015). They are further subdivided into two subfamilies, the Lemnoideae (*Spirodela, Landoltia*, and *Lemna*) and Wolffioideae (*Wolffiella* and *Wolffia*) (Les et al., 2002), the latter being the rootless duckweeds. Fast-growing and starch accumulation capacities are some of the main features of duckweed, with some strains having been shown to double in biomass within 96 h (10 times faster than maize) (Yu et al., 2014).

Plant growth depends on carbon assimilation through photosynthesis. During the day, starch is synthesized and stored in the plastids, whereas sucrose is stored in vacuoles or directly used for growth. In the dark, the stored starch will serve as the main compound to support plant growth (Mengin et al., 2017). Thus, starch contents vary according to photoperiod (Smith and Stitt, 2007; Zeeman et al., 2007, 2010; Fernandez et al., 2017). Yin et al. (2015) found positive correlations between day length, light intensity, and level of starch accumulation in *Lemna aequinoctialis*. Starch levels are also found to be related to the nutrient status (Xiao et al., 2013). These authors found that growth of *Landoltia punctata, Spirodela polyrhiza*, and *L. aequinoctialis* is boosted with a concomitant decrease of starch under higher availability of P and N. Thus, lower concentrations of starch can be a sign for higher growth rates and vice-versa.

In plants, most of the carbon assimilated by photosynthesis is partitioned into cell walls (Vaughan et al., 1992; Verbančič et al., 2017). Plant cell walls form a Glycomic Code (Buckeridge, 2018) that may help to determine their structure-function relationship. The cell wall is composed of polysaccharides, phenolic compounds, and proteins, the former being quantitatively more dominant. Besides providing mechanical support for plant tissues, the cell wall also acts as a defense mechanism by presenting a physical barrier to biological invaders (Sarkar et al., 2009; Kalluri and Keller, 2010). In cell walls, cellulose microfibrils are the architectural core to which hemicelluloses (xyloglucan, arabinoxylans, mannans, beta-glucans, and others) are attached. This domain (cellulose-hemicellulose) is immersed in a matrix of pectins that include homogalacturonans and rhamnogalacturonans, branched with neutral chains of galactans, arabinans, and arabinogalactans (Carpita and Gibeaut, 1993). In this regard, the pectin domain of many duckweeds is unique. Besides rhamnogalacturonans and arabinogalactans (Venketachalam et al., 2013), many species of the Lemnaceae have been found to have cell walls enriched with apiogalacturonan, a pectin polymer rich in apiose (Hart and Kindel, 1970; Mølhøj et al., 2003; O'Neill et al., 2004; Camacho-Cristóbal et al., 2008; Miwa and Fujiwara, 2010; Bar-Peled and O'Neill, 2011). Recently, Avci et al. (2018) observed that pectins in Lemnoideae are mainly apiogalacturonans, whereas in Wolffioideae the apiogalacturonan content is reduced, and replaced by xylogalacturonan. The authors highlighted a possible evolutionary trend in duckweeds associated with species-dependent variations in apiogalacturonan and xylogalacturonan (Avci et al., 2018). Apiose-containing polysaccharides are thought to play a role in the boron binding capacity of duckweeds (Matoh and Kobayashi, 1998) as well as to plant development and growth (Blevins and Lukaszewski, 1998; Matoh and Kobayashi, 1998). Relatively little is known about the other cell wall polymers of duckweeds (Zhao et al., 2014). Hemicelluloses have been reported to be in small amounts (3%) in duckweed cell walls (Ge et al., 2012; Zhao et al., 2014), consistent with the presence of very low lignin levels in duckweed biomass (Blazey and McClure, 1968). Venketachalam et al. (2013) identified xyloglucans and xylans in the glycome profile of *Lemna*, while cellulose has been reported to be present at 43.7% in *Lemna minor* cell walls (Zhao et al., 2014)

In this work, we performed a comparative analysis of growth rates and carbohydrate contents under different light conditions with five species of duckweed from the different genera. We found that under higher light intensity, a condition in which the RGR increases in all species, the faster-growing Lemnoideae species accumulate less starch than the slower-growing Wolffioideae species. At the same time, the higher light intensity increased growth as well as the proportion of apiose in the cell walls of Wolffioideae. Our results suggest that the presence of apiose-containing polymers in cell walls of duckweeds could be related to the growth capacity of duckweed species.

## Materials and methods

### Plant material, cultivation, and sample preparation

**Figure 1:**
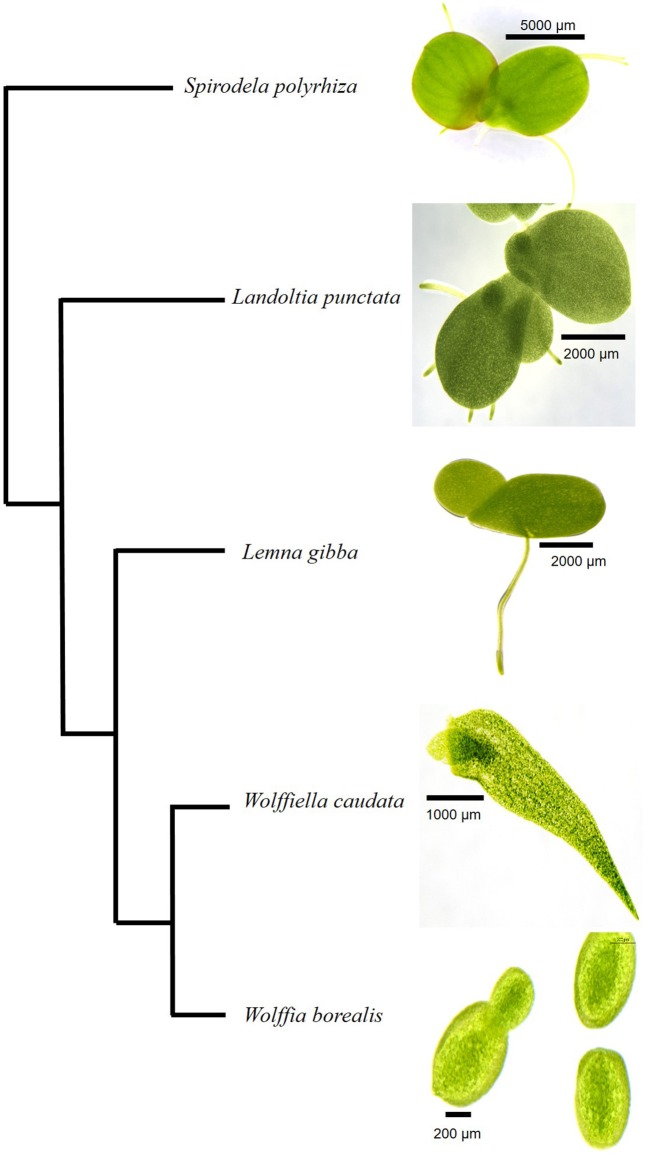
Cladogram of *Lemnaceae* species analyzed in the present study. The five species from different genera of duckweeds are shown according to the phylogeny suggested by Tippery et al. (2015). A size bar in each photograph indicates the scale as shown for the different specimen.

*Spirodela polyrhiza* (9509), *Landoltia punctata* (7624)*, Lemna gibba* (DWC128)*, Wolffiella caudata* (9139), and *Wolffia borealis* (9144) were obtained from the Rutgers Duckweed Stock Cooperative (RDSC) collection (Figure 1). Duckweeds were cultivated under axenic conditions in 100 mL of $\frac{1}{2}$ Schenk-Hildebrandt medium (pH 6.5) with 0.5% of sucrose. The plants were grown at 25°C with a photoperiod of 16 h of light. Two different light intensities were used: 20 μmoles m^−2^ s^−1^ and 500 μmoles m^−2^ s^−1^. The cultivated plants were frozen in liquid nitrogen and freeze-dried. The freeze-dried samples were transferred to 15 mL polycarbonate vials with the screw-on cap including a steel grinding ball of 11 mm that was placed in Geno/Grinder®2010 SPEX SamplePrep for sample processing at 1400 rpm for 2 min or until a fine powder achieved.

### Relative growth rate measurements

The RGR parameters were calculated according to International Steering Committee on Duckweed Research and Applications (ISCDRA). The growth measurements followed the procedure described by Ziegler et al. (2015). Twenty fronds of *S. polyrhiza, L. punctata, L. gibba*, and 50 fronds from *W. caudata* and *W. borealis* were initially inoculated into the culture medium. At the same time, for T_0_ (the initial point of analysis) four replicates were harvested for measurements of dry mass. T_7_ was harvested after 7 days of growth. Samples were dried at 105°C for 12 h and weighed to obtain dry mass. RGR was calculated by equation I that was simplified into equation II, where x represents the data of the evaluated parameters (dry mass and number of fronds) and t represents elapsed time (zero -*t*_0_-and 7 days-*t*_7_).

$$\begin{array}{l} X_{t}=x_{t0}*e^{RGR*t}\;\left(I\right)\\ RGR=\frac{lnx_{t7}-lnx_{t0}}{t_{7}-t_{0}}\;\left(II\right) \end{array}$$

### Soluble carbohydrate extraction and analysis

Soluble sugars (glucose, fructose, sucrose, and raffinose) were extracted four times from 20 mg (dry mass) of pulverized samples with 1.5 mL of 80% ethanol at 80°C for 20 min. The Alcohol Insoluble Residue (AIR) was dried at 45°C overnight. The supernatant was recovered, vacuum concentrated (ThermoScientific® Savant SC 250 EXP) and resuspended in 1 mL of water and 1 mL of chloroform. The soluble sugars (sucrose, fructose, glucose, and raffinose) were analyzed by High-Performance Anion Exchange Chromatography with Pulsed Amperometric Detection (HPAEC-PAD) in a Dionex® system (ICS 5000) using a CarboPac PA1 column and eluted with 150 μM sodium hydroxide in an isocratic run of 27 min (Supplementary Figures 1, 2).

### Starch removal and determination

Starch was measured according to Amaral et al. (2007) and Arenque et al. (2014). AIR was treated with 120 U/mL of α-amylase (E.C. 3.2.1.1) of *Bacillus licheniformis* (Megazyme® Inc., Australia) diluted in 10 mM MOPS buffer pH 6.5 at 75°C for 1 h. Incubation was followed by addition of 30 U/mL of amyloglucosidase (E.C. 3.2.1.3) of *Aspergillus niger* (Megazyme® Inc., Australia) diluted in 100 mM sodium acetate pH 4.5 at 50°C for 1 h. The reactions were stopped by freezing the samples. The supernatants were recovered by centrifugation, and the pellets were washed three times with 80% ethanol and dried at 45°C overnight and reserved for monosaccharides analysis. For starch determination, 5 μL of each sample was diluted with 45 μL of deionized water followed by 250 μL of a mixture containing glucose oxidase (1,100 U/mL), peroxidase (700 U/mL), 4-aminoantipirin (290 μmol/L) and 50 mM of phenol at pH 7.5. The plates were incubated for 15 min at 30°C and the absorbance was measured at 490 nm. The calibration curve was performed with commercial glucose (Sigma®) in the concentration range of 0.02–0.2 mg/mL.

### Monosaccharide composition

Five mg of the cell wall (de-starched AIR) was hydrolyzed with 1 mL of 2 M trifluoroacetic acid (TFA) for 1 h at 100°C. The reaction mixture was dried under vacuum and resuspended in 1 mL of deionized water. This was followed by filtration on 0.22 μm (Merck Millipore®) filters. The released monosaccharides were analyzed by HPAEC-PAD through the injection of 10 μL hydrolysate into a CarboPac SA10 column (ICS 5.000 system, Dionex-Thermo®). The column was eluted isocratically with 99.2% of water and 0.8% (v/v) sodium hydroxide (1 mL/min). The monosaccharide release from the cell wall were detected using a post-column base containing 500 mM NaOH (0.5 mL/min). The standards used were apiose, arabinose, fucose, galactose, glucose, mannose, rhamnose, and xylose (Supplementary Figure 1). Quantification was performed by injections of samples with known concentrations for each monosaccharide to calibrate the instrument.

### Uronic acid determination

The total uronic acid was quantified according to Filisetti-Cozzi and Carpita (1991). Five mg of each de-starched cell wall were weighed and 2 mL of concentrated sulfuric acid were added. The reactions were incubated for 10 min on ice under stirring (1,250 rpm), followed by addition of 1 mL of deionized water. This procedure was repeated once. The incubated mixtures were diluted to 10 mL and centrifuged at 4,000 g for 10 min at room temperature. Forty μL of 4 M sulfamic acid/potassium sulfamate solution (pH 1.6) and 2.4 mL of 75 mM sodium borate in sulfuric acid was added to aliquots of supernatant (400 μL). The homogenized solutions were incubated at a 100°C for 20 min, then cooled on ice for 10 min. Eighty microliter of m-hydroxybiphenyl in 0.5% NaOH were added and vortexed for color development. The samples were read at 525 nm in Spectrophotometer Genesys 10S UV-VIS ThermoScientific®. A standard curve using D-galacturonic acid was performed in the concentration range of 5–40 μL/400 μL.

### Statistical analysis

Four replicates were used for the experiments. Interspecific analyses were performed by ANOVA one-way followed by Tukey's test (*p* < 0.05). A *t*-test was used to compare different light conditions. The analyses were carried out using JMP® software version 5.1 or R version 3.2.2. Principal Component Analysis was performed using Minitab software version 14 with all data for low light and high light treatments. General Linear Model (GLM) was used to evaluate the significance of each principal component (Supplementary Table 2).

## Results

### Relative growth rates

**Figure 2:**
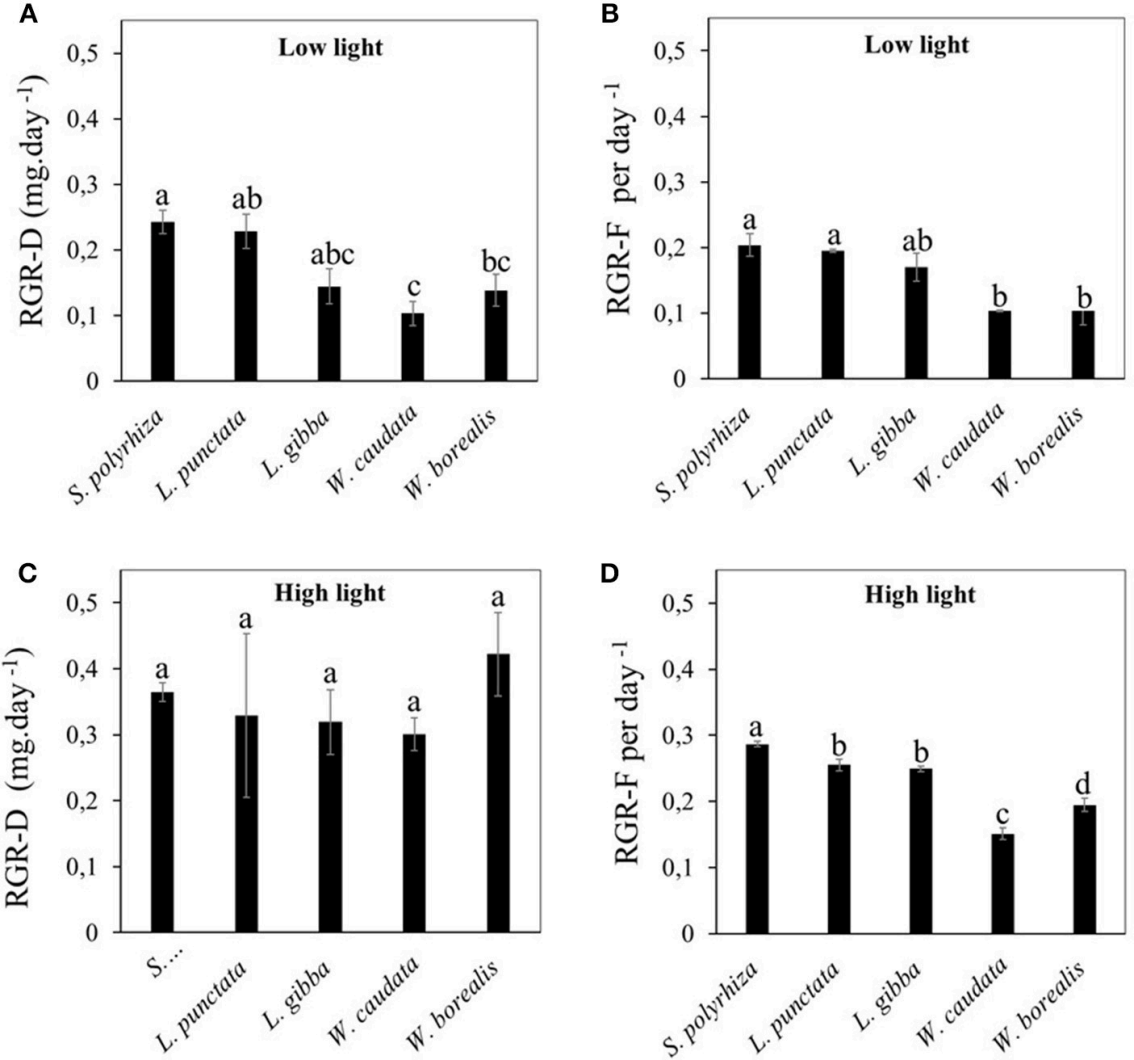
Comparison of relative growth rate (RGR) for different duckweed strains (*S. polyrhiza, L. punctata, L. gibba, W. caudata*, and *W. borealis*) cultivated under two light intensities (20 μmol.m^−2^.s^−1^ and 500 μmol.m^−2^.s^−1^). Values are average ± standard error evaluated per dry mass **(A,C)** and the number of fronds **(B,D)**. **(A,B)** show data from low light condition while **(C,D)** represent data from high light condition. Means followed by the same letters are statistically significant among the five species compared according to Tukey's test (*p* < 0.05).

We first compared the RGR between the five species of duckweed under illumination at intensity of 20 μmoles m^−2^ s^−1^. Using either dry weight (RGR-D, Figure 2A) or frond number (RGR-F, Figure 2B) as the basis for RGR calculation, we found that the rates for the two Wolffioideae species (*W. caudata* and *W. borealis*) were significantly lower (0.1–0.14 day^−1^) than the other species of the Lemnoideae (0.14–0.24 day^−1^). In contrast, when compared under higher light intensity of 500 μmoles m^−2^ s^−1^, RGR-F measurements continue to show lower growth rates for the Wolffioideae (Figure 2D) while RGR-D measurements showed equally high growth rates for all five species of duckweed (Figure 2C).

Quantifying the effects of light intensity on these parameters for each species revealed that RGR-D increased the most for the Wolffioideae under higher light intensities, being at around 50% for *S. polyrhiza* (53.9%) and *L. punctata* (42.7%) and more than doubling for *L. gibba* (147.9%), *W. caudata* (203.4%), and tripling for *W. borealis* (218.6%). Since the frond number increase (RGR-F) in the Wolffioideae continues to be below that of the Lemnoideae (Figure 2D), our results indicate that the fronds of Wolffioideae produced under higher light must be higher in dry mass than the Lemnoideae fronds, thus resulting in similar RGR-D under high light (Figure 2C). One possible cause for this would be a higher starch content in the Wolffioideae than the Lemnoideae under this condition.

### Duckweed carbohydrates

**Figure 3:**
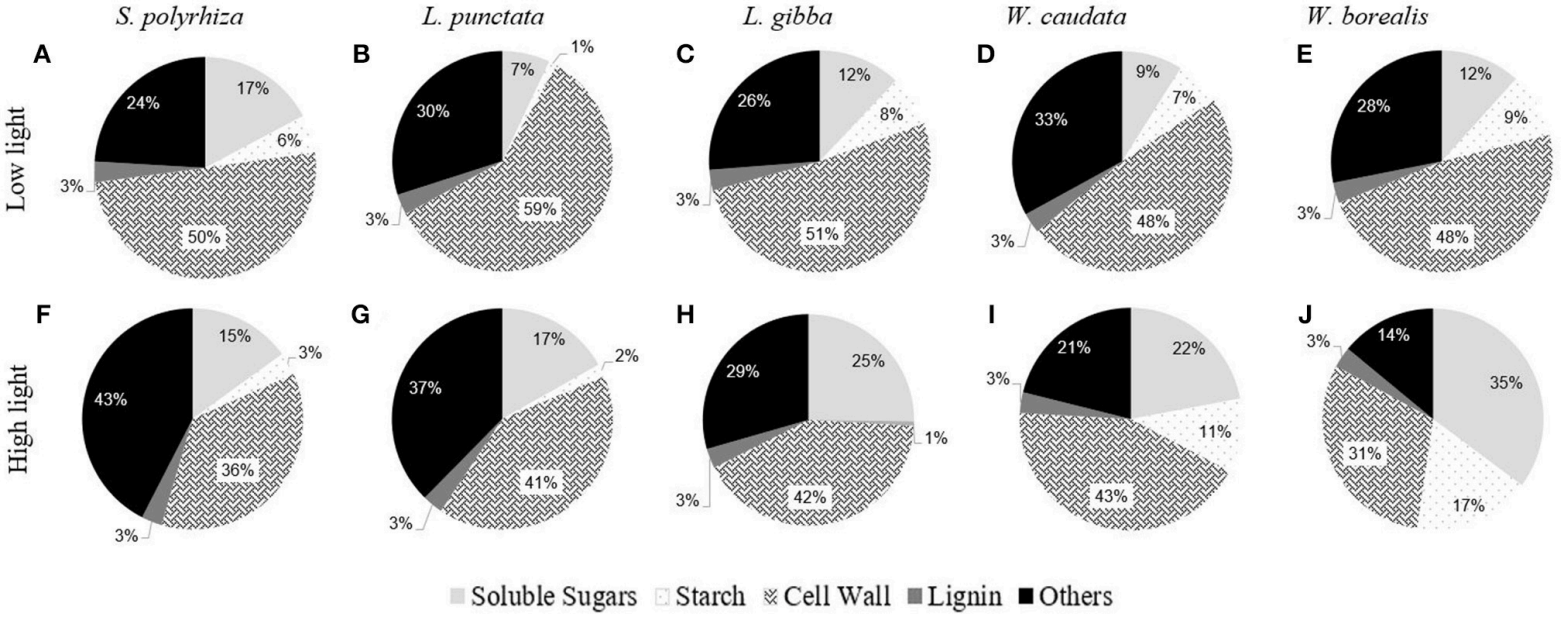
Comparative composition of duckweeds (*S. polyrhiza, L. punctata, L. gibba, W. caudata*, and *W. borealis*) biomass cultivated under low light (20 μmol.m^−2^.s^−1^) and high light (500 μmol.m^−2^.s^−1^) intensity. Values are averages from the percentages of dry mass. Soluble sugars are the sum of the contents of sucrose, fructose, raffinose, and glucose. Cell walls are the residual portion of de-starched AIR. (*n* = 4). The capital letters represent the species and the light treatment. **(A–E)** are the biomass composition of plants cultivated under low light and **(F–J)** under high light.

Figure 3 shows a comparison of the carbohydrate composition for each species of duckweed grown under low (20 μmoles m^−2^ s^−1^) and high light intensity (500 μmoles m^−2^ s^−1^) conditions. Two different statistical approaches were taken. One compares the differences among species (ANOVA One-way) under the two light conditions (Supplementary Table 1), and the other compares effects of light on each species, using a *t*-test for the two light treatments (Table 1). With respect to the species studied in this work, light intensity had relatively small effects on carbohydrate partitioning in terms of starch and soluble sugars (Figure 3, Supplementary Table 1). On average, accounting only for the carbohydrates, soluble sugars represented 17.1%, starch 6.4%, and cell walls 47.9% of the biomass (Figure 3, Supplementary Table 1). Other components such as lipids, proteins, and secondary metabolites accounted for about 28.6% (Figure 3). As reported by previous workers, we found a constant low level of 3% lignin in these aquatic plants, irrespective of the light conditions and relative growth rates (data not shown).

**Table 1:** Sugar composition of duckweeds (*S. polyrhiza, L. punctata, L. gibba, W. caudata*, and *W. borealis*) biomass (μg.mg^−1^ dry mass) cultivated under low light (20 μmol.m^−2^.s^−1^) (LL) and high light (500 μmol.m^−2^.s^−1^) (HL) intensities.

		***S. polyrhiza***	***L. punctata***	***L. gibba***	***W. caudata***	***W. borealis***
**NON-STRUCTURAL CARBOHYDRATES (**μ**g.mg**^−1^ **DM)**						
Glucose	LL	5.03 ± 2.11	2.64 ± 0.40	6.10 ± 2.19	4.00 ± 0.45	5.12 ± 1.04
	HL	2.66 ± 0.19	1.63 ± 0.17	4.54 ± 0.55	2.40 ± 0.25	12.38 ± 4.84
	*P*-value	0.306	0.061	0.515	**0.020**	0.193
Fructose	LL	6.51 ± 3.01	4.33 ± 0.72	5.67 ± 2.14	3.99 ± 0.41	6.37 ± 1.19
	HL	2.89 ± 0.32	1.84 ± 0.19	5.80 ± 0.72	5.19 ± 0.87	17.03 ± 5.83
	*P*-value	0.276	**0.015**	0.953	0.259	0.124
Sucrose	LL	5.47 ± 2.21	0.04 ± 0.01	0.08 ± 0.02	0.06 ± 0.01	0.17 ± 0.02
	HL	8.81 ± 0.65	6.48 ± 0.50	11.02 ± 0.58	14.26 ± 2.25	5.66 ± 0.22
	*P*-value	0.198	**0.000**	**0.000**	**0.001**	**0.000**
Raffinose	LL	0.22 ± 0.10	0.01 ± 0.01	0.21 ± 0.11	0.89 ± 0.12	0.02 ± 0.01
	HL	0.68 ± 0.08	6.90 ± 0.51	3.85 ± 0.53	0.19 ± 0.06	0.12 ± 0.03
	*P*-value	**0.011**	**0.000**	**0.001**	**0.002**	**0.018**
Starch	LL	55.23 ± 13.88	11.97 ± 0.69	74.04 ± 23.61	66.49 ± 18.77	94.71 ± 21.94
	HL	32.95 ± 1.98	18.56 ± 3.19	6.88 ± 0.21	106.88 ± 20.61	171.54 ± 27.67
	*P*-value	0.163	0.090	**0.029**	0.198	0.073
**STRUCTURAL CARBOHYDRATES (**μ**g.mg**^−1^ **DM)**						
Uronic acids	LL	184.96 ± 17.64	66.98 ± 5.85	169.86 ± 18.18	180.36 ± 17.53	226.82 ± 33.34
	HL	127.57 ± 12.73	174.51 ± 18.79	143.77 ± 16.41	140.65 ± 36.66	123.24 ± 14.33
	*P*-value	**0.039**	**0.002**	0.061	0.045	0.147
Fucose	LL	1.54 ± 0.12	1.19 ± 0.21	0.98 ± 0.06	1.06 ± 0.06	1.42 ± 0.16
	HL	1.14 ± 0.06	0.53 ± 0.13	0.67 ± 0.02	1.25 ± 0.25	1.74 ± 0.27
	*P*-value	**0.026**	**0.040**	**0.003**	0.497	0.343
Apiose	LL	7.27 ± 0.90	19.47 ± 3.61	15.80 ± 0.75	3.61 ± 0.71	1.94 ± 0.27
	HL	5.42 ± 0.34	14.64 ± 0.72	10.16 ± 1.13	7.00 ± 1.85	3.23 ± 0.36
	*P*-value	0.103	0.238	**0.006**	0.139	**0.030**
Xylose	LL	8.47 ± 0.38	23.20 ± 4.48	18.26 ± 0.73	13.92 ± 0.78	15.98 ± 0.65
	HL	6.94 ± 0.31	12.63 ± 4.03	14.57 ± 1.86	24.33 ± 5.39	27.19 ± 4.20
	*P*-value	**0.020**	0.130	0.115	0.104	**0.039**
Arabinose	LL	11.57 ± 1.02	10.34 ± 1.91	6.17 ± 0.15	28.76 ± 4.72	14.46 ± 0.65
	HL	9.78 ± 0.71	4.03 ± 1.10	5.64 ± 0.48	39.05 ±7.02	23.80 ± 3.23
	*P*-value	0.203	**0.028**	0.336	0.270	**0.030**
Galactose	LL	13.80 ± 0.77	12.78 ± 2.62	8.91 ± 0.20	9.96 ± 0.89	8.68 ± 0.73
	HL	11.63 ± 0.81	6.96 ± 2.22	8.35 ± 0.61	11.60 ± 2.19	11.61 ± 1.87
	*P*-value	0.101	0.142	0.420	0.516	0.196
Rhamnose	LL	3.88 ± 0.24	3.26 ± 0.70	2.36 ± 0.08	1.72 ± 0.14	2.40 ± 0.21
	HL	3.23 ± 0.18	1.69 ± 0.53	1.88 ± 0.17	2.31 ± 0.58	2.37 ± 0.33
	*P*-value	0.073	0.124	**0.050**	0.364	0.950
Glucose	LL	3.39 ± 0.44	3.05 ± 0.45	3.02 ± 0.71	5.07 ± 0.81	4.74 ± 1.46
	HL	9.02 ± 1.08	2.96 ± 0.92	4.76 ±1.30	14.27 ± 3.49	17.96 ± 3.95
	*P*-value	**0.003**	0.932	0.284	**0.043**	**0.020**
Manose	LL	1.95 ± 0.16	8.63 ± 1.78	0.91 ± 0.07	1.35 ± 0.17	1.76 ± 0.21
	HL	1.50 ± 0.26	0.91 ±0.26	1.15 ± 0.08	1.42 ± 0.30	1.55 ± 0.28
	*P*-value	0.195	**0.005**	0.061	0.847	0.568

*Data are given as averages ± standard errors. P-values (P < 0.05) that are statistically significant are presented in bold (n = 4)*.

Table 1 shows the carbohydrate composition of the five species of duckweeds used in this work. Under high light, sucrose and raffinose increased in most of the species studied, while fructose and glucose decreased in the Lemnoideae and increased in Wolffioideae. An exception was *W. caudata*, for which glucose and raffinose decreased significantly under high light (Table 1). Statistical differences could be seen between Lemnoideae and Wolffioideae under high light treatment, with induction of starch accumulation in Wolffioideae (Supplemental Table 1 and Table 1). The analysis of cell wall components under the two light treatments revealed the following trends upon increase in light intensity: (1) uronic acids decreased for most species except for *L. punctata*, where the main features were a significantly lower uronic acids and a higher mannose content in low light in comparison with the other species; (2) all the monosaccharides except for mannose and glucose decreased in Lemnoideae species whereas we observed an increase in most of them in Wolffioideae.

**Figure 4:**
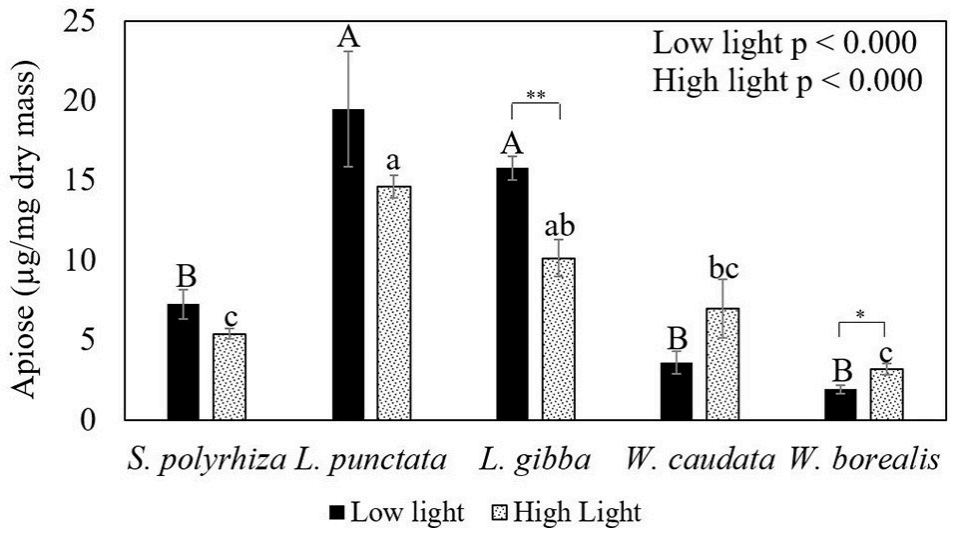
Comparative analysis of the apiose content in the cell walls of duckweeds (*S. polyrhiza, L. punctata, L. gibba, W. caudata*, and *W. borealis*) cultivated under two light intensity (20 and 500 μmol photons m^−2^ s^−1^) conditions. Data are the averages ± standard errors (*n* = 4). Letters in capital on top of darker bars are the significant differences by Tukey's test (*p* < 0.05) for low light. Lowercase letters on top of gray bars mean significant differences regarding high light. Asterisks indicate significant differences by *t*-test (*p* < 0.05) for species in two light intensities.

Figure 4 and Table 1 show the trends in apiose in the cell walls for the species studied. There is a significant trend toward higher apiose content in Lemnoideae in comparison with Wolffioideae. Furthermore, under higher light intensity, there was a trend toward a decrease in apiose for Lemnoideae (significant for *L. gibba*) in contrast to the inverse trend toward higher apiose observed with the two Wolffioideae species (more significantly for *W. borealis*) (Figure 4). The apiose content evaluated was only structural, found in the pectin fractions.

**Figure 5:**
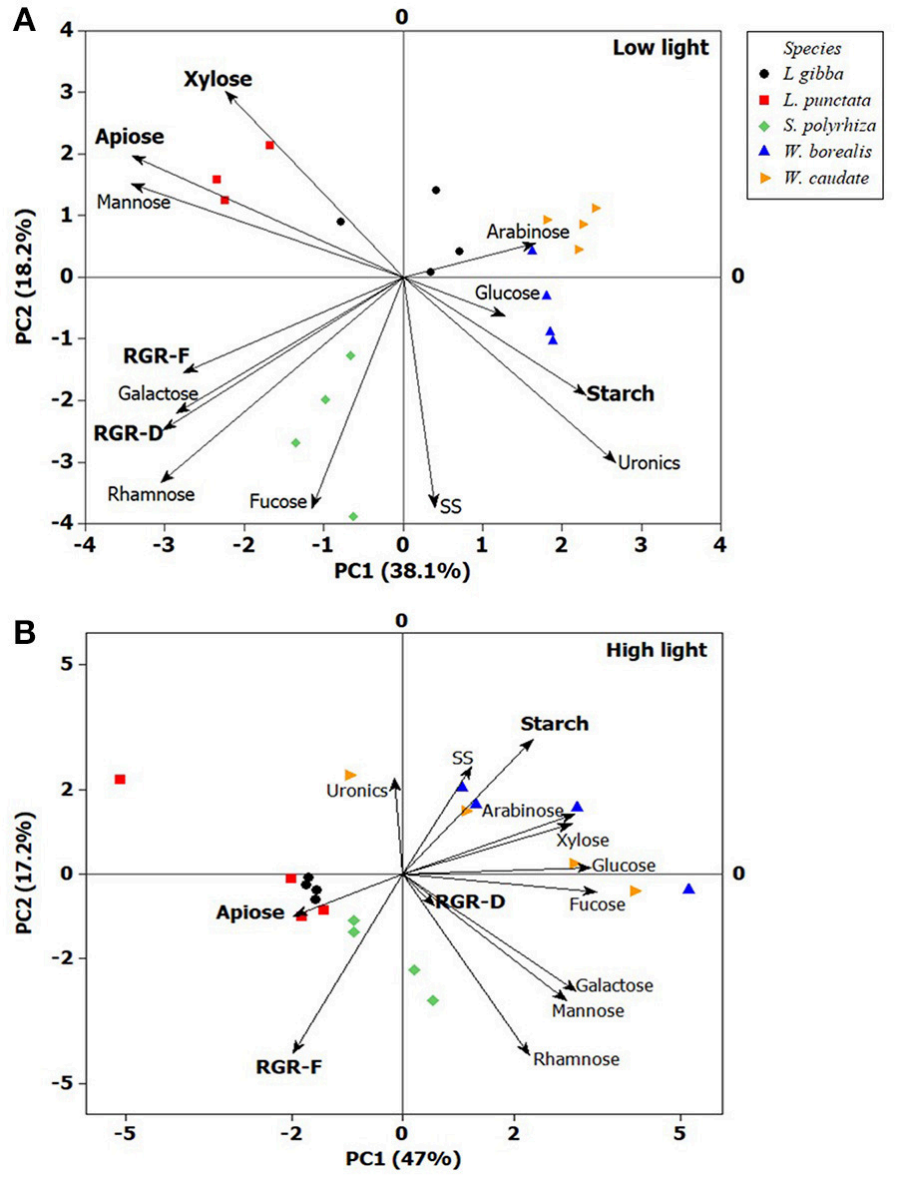
Principal Component Analysis (PC1 and PC2) of duckweeds (*S. polyrhiza, L. punctata, L. gibba, W. caudata*, and *W. borealis*) grown under two light intensities. **(A)** low light (20 μmol photons m^−2^ s^−1^) and **(B)** high light (500 μmol photons m^−2^ s^−1^). Xylose, apiose, mannose, arabinose, glucose, galactose, and rhamnose are cell wall-related monosaccharides, uronic acids (uronics) is a component of pectin and some hemicelluloses, starch and SS (Soluble Sugars) represent non-structural carbohydrates, and RGR (Relative Growth Rate) based on number of fronds (RGR-F) and dry mass (RGR-D). The vector values and statistical analyses are in the Supplementary Table 2. (*n* = 4).

Principal Component Analysis (Figure 5) using all the variables confirmed the observations above and revealed that starch and apiose contents are negatively correlated. In the case of low light, this can be explained by the higher capacity of growth with *S. polyrhiza* (Figure 5A), which could be the reason for its clear separation from all the other species tested (PC2). However, under high light (Figure 5B), the species from Lemnoideae, which has higher apiose content, propagated faster (RGR-F), whereas the Wolffioideae species grew slower (RGR-F), concomitant with accumulating higher amounts of starch.

## Discussion

In this study, we found significant differences between Lemnoideae and Wolffioideae regarding growth, starch content and cell wall components. For the most part, both subfamilies of duckweeds follow the pattern known for most plant species, i.e., that lower starch content is correlated with higher growth rates (Schulze et al., 1991; Sulpice et al., 2009; Zeeman, 2015). However, our study revealed that Lemnoideae apparently differs from Wolffioideae by displaying higher growth rates with relatively low starch contents under high light condition, while *W. borealis* and *W. caudata* (Wolffioideae) display growth limitation under higher light conditions with a concomitant increase in starch accumulation (Figures 2C,D). Our results suggest that the increase in biomass of Wolffioideae under high light is not solely due to an increase in the number of fronds, but may be driven largely by the fact that the fronds become heavier due to the accumulation of starch (Table 1, Supplementary Table 1). Thus, there seems to be a limitation for growth that is unrelated to light quantity and possibly associated with the internal control of carbon allocation and/or photosynthetic capacity. It is important to note that non-structural carbohydrates may vary considerably in duckweeds growing in the wild. Indeed, Xiao et al. (2013) compiled data and found that starch in duckweeds can vary from 3 to 75%. The authors concluded that this variation is probably due to geographical location and nutritional factors. Despite the fact that variations in the wild can occur, this is not valid for the interpretation of our results, since the species used in this work were cultivated under the same controlled conditions.

Duckweed biomass has been reported to display higher proportions of soluble compounds, which contrasts with the relatively lower proportions of structural carbohydrates (Xu et al., 2011; Xiao et al., 2013; Yin et al., 2015; Li et al., 2016). However, our study revealed that structural carbohydrates make up almost half of the biomass in all species analyzed (Figure 3). The cell wall of duckweeds has been thought to display higher percentages of pectin (Avci et al., 2018). Duff (1965) was the first to report apiose occurrence in duckweed. This five-carbon sugar was found by Hart and Kindel (1970) as a major constituent of apiogalacturonan in duckweeds (*Lemna minor*). More recently, Avci et al. (2018) determined the structure of several pectic polysaccharides (rhamnogalacturonans, apiogalacturonan, and xylogalacturonan) from 12 species of duckweed and found that the ones from the subfamily Wolffioideae contain lower proportions of apiogalacturonan. These authors also highlight that there is a trade-off between apiogalacturonan and xylogalacturonan in duckweeds, with the latter polysaccharide being higher in Wolffioideae. Xylose can be found in xylans, xyloglucan, arabinoxylans, rhamnogalacturonan, and xylogalacturonan (Guyett et al., 2009; Bar-Peled et al., 2012). This sugar is reported to be lower in duckweeds (Ge et al., 2012; Zhao et al., 2014). However, in the present study, we found that xylose represented approximately 22% of the cell wall (82.84 μg mg^−1^ DM), decreasing in Lemnoideae (0.68 times) and increasing in Wolffioideae (1.7 times) with an increase in light intensity during growth (Table 1, Figure 5). It would be interesting to examine the molecular basis for this variation and the biochemical consequences in terms of cell wall architectural differences between these subfamilies. The recently reported high-quality reference genome for *S. polyrhiza* (Michael et al., 2017) and the soon-to-be completed *Wolffia australiana* genome (E. Lam, unpublished work) may help to shed light on this aspect.

It has been hypothesized that the presence of apiose (i.e., apiogalacturonans) in duckweeds can be related to their expected need to efficiently assimilate boron (Matoh, 1997) due to the known ability of apiose to specifically form complexes with boron in plant cell walls (Fleischer et al., 1998; Matsunaga et al., 2004). While apiose is present in rhamnogalacturonan II of plants, the existence of apiogalacturonan appears to be unique to aquatic plants such as duckweed (Hart and Kindel, 1970; Mohnen, 2008). We thus hypothesize that the presence of significant amounts of apiogalacturonan in duckweed cell walls could be an evolutionary adaptation of this family of aquatic plants to thrive in their habitat. Since aquatic environments usually have a concentration of boron that is 300 times lower than the average in terrestrial environments (Power and Woods, 1997; Shorrocks, 1997; Lemarchand et al., 2000), it would be reasonable to think that the concentration of apiogalacturonan in the cell walls could be an adaptive factor related to their rapid growth on the water surface.

In addition to the potential relationship with boron adsorption, it is also known that reduced cross-links in rhamnogalacturonan II are related to decreased plant growth (Ishii et al., 2001). The recent report that the structure and cross-linking of this polysaccharide in Lemnoideae and Wolffioideae is conserved (Avci et al., 2018) further strengthens the hypothesis for the positive contribution of apiogalacturonans to enhanced duckweed growth.

In the present study, a trade-off between the accumulation of starch and apiose (probably apiogalacturonnan) has been observed, with higher starch and low apiose levels in Lemnoideae and the reverse trend in Wolffioideae. This was found for 4 out of the 5 species studied. The exception of this was *L. punctata*, in which starch did not vary under different light intensities and uronic acids were rather low under low light when compared to the other evaluated species. A possible explanation for these findings (see Supplementary Figure 3 for some of the possible metabolic pathways present in duckweeds) is that the light intensity used in our experiment was far too low for *L. punctata* in comparison to the other species studied here. This led to a carbon flow that, although still supporting some growth, did not afford cells to establish two of their substantial carbohydrate sinks (starch and pectins). The absence of an increase in starch in the non-structural carbohydrate pool, at the same time as the comparatively low uronic acids levels in the cell walls of *L. punctata*, is consistent with the hypothesis that *L. punctata* may have rechanneled carbon metabolism toward the GDP-mannose pathway under low light. In fact, this hypothesis is corroborated by the observation of a 10-fold higher accumulation of mannose in this species in comparison with *W. caudata* (Table 1).

More apiose was found in the species of Wolffioideae cultivated under high light, with a concomitant increase in their growth rates. Nevertheless, their growth rates in terms of development (RGR-F) were still lower and their starch content higher when compared to those of the three Lemnoideae (except for *L punctata* in this work) species studied under the same conditions. In the Lemnoideae species, where apiose is usually high even when grown under low light intensity, their higher growth rates were consistent with lower accumulation of starch as well.

In sum, our results suggest that apiogalacturonans in duckweeds may be a rate-limiting factor for growth of duckweeds, especially in species belonging to the subfamily of Wolffioideae. If proven true in further screening of additional species and accessions in this subfamily of duckweeds, the levels of apiose and apiogalacturonans may be useful biomarkers for identification of high growth rate duckweed strains for commercial applications.

## Author contributions

MB and EL planned the work, DP and EI performed the experiments, DP and AG analyzed the data, MB, AG, DP, and EL wrote the manuscript.

### Conflict of interest statement

The authors declare that the research was conducted in the absence of any commercial or financial relationships that could be construed as a potential conflict of interest.
