# Supplementary material for: Correlation of Apiose Levels and Growth Rates in Duckweeds
Source: Front Chem. 2018 Jul 20;6:291. doi: 10.3389/fchem.2018.00291 (PMC6062639; doi:10.3389/fchem.2018.00291)

Supplementary Material

Correlation of Apiose Levels and Growth Rates in Duckweeds

Débora Pagliuso, Adriana Grandis, Eglee Sílvia Igarashi, Eric Lam, Marcos Silveira Buckeridge*

*** Correspondence: Eric Lam: eric.lam@rutgers.edu;** Marcos Silveira Buckeridge: msbuck@usp.br

**
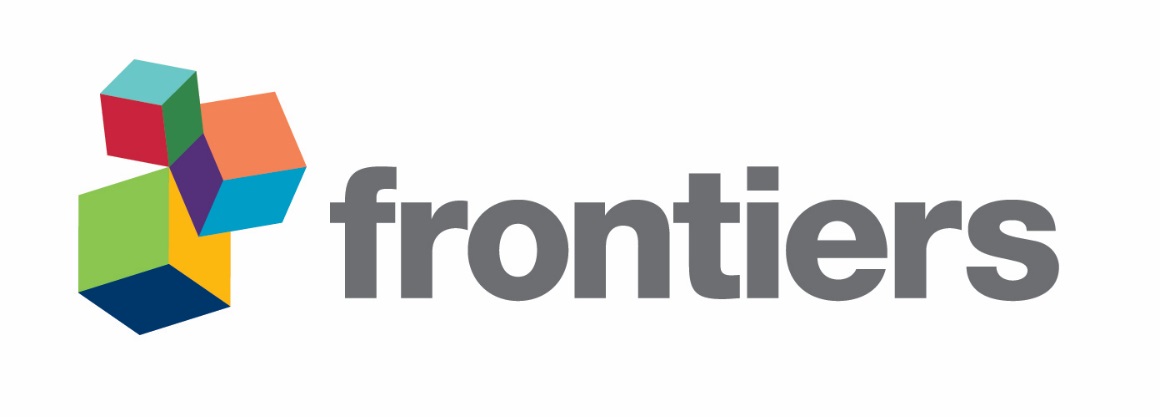
**

**Supplementary Table 1.** **Analysis for duckweed biomass composition data (from Figure 3).** Values of the columns represent a standard mean ± standard error (n=4). Statistics analysis were performed with ANOVA one-way for each light intensity and sugar separately (p-value is shown). The variance analysis followed Tukey´s comparison of means per sugar (column) with highlighted letters in bold fonts indicate the significant values in confidence interval of 95% or more.

| **Treatment** | **Species** | **% Cell wall** | **% Soluble sugars** | **% Starch** | **%Others** |
| --- | --- | --- | --- | --- | --- |
| Low light | *S. polyrhiza* | 50.16 ± 1.64^b^ | 17.24 ± 4.26 | 5.52 ± 1.38^ab^ | 27.07 ± 5.59 |
|  | *L. punctata* | 58.84 ± 0.80^a^ | 7.02 ± 1.08 | 1.19 ± 0.07^b^ | 32.93 ± 1.89 |
|  | *L. gibba* | 51.33 ± 1.76^b^ | 12.05 ± 4.38 | 7.40 ± 2.36^ab^ | 29.21 ± 3.18 |
|  | *W. caudata* | 48.42 ± 17.4^b^ | 8.85 ± 0.98 | 6.65 ± 1.88^ab^ | 35.97 ± 2.17 |
|  | *W. borealis* | 47.74 ± 0.63^b^ | 11.68 ± 2.26 | 9.47 ± 2.19^a^ | 31.10 ± 1.94 |
|  | *p-value* | **0.000** | 0.209 | **0.054** | 0.373 |
| High light | *S. polyrhiza* | 36.23 ± 0.84 | 15.04 ± 1.12 | 3.29 ± 0.19^b^ | 45.43 ± 1.59^b^ |
|  | *L. punctata* | 40.67 ± 1.72 | 16.85 ± 1.35 | 1.85 ± 0.32^b^ | 40.61 ± 1.41^ab^ |
|  | *L. gibba* | 41.67 ± 1.41 | 25.21 ± 2.36 | 0.68 ± 0.02^b^ | 32.43 ± 2.49^abc^ |
|  | *W. caudata* | 43.16 ± 7.18 | 22.03 ± 3.34 | 10.69 ± 2.06^a^ | 24.11 ± 6.14^bc^ |
|  | *W. borealis* | 30.63 ± 3.38 | 35.19 ± 10.82 | 17.53 ± 2.76^a^ | 17.02 ± 5.59^c^ |
|  | *p-value* | 0.164 | 0.103 | **0.000** | **0.000** |

**Supplementary Table 2. Statistics and variance proportions corresponding to axis (PC1 and PC2) generated by Principal Component Analysis from sugars and growth rate (RGR) values for five species of duckweeds.** SS: soluble sugars, RGR-D: relative growth ratio based upon dry mass, RGR-F: relative growth ratio based upon number of fronds, others (starch, arabinose, fucose, glucose, mannose, rhamnose, xylose, apiose and uronic) are carbohydrates measured (n=4).

|  | **Low light** | |  | **High Light** | |
| --- | --- | --- | --- | --- | --- |
|  | **PC1** | **PC2** |  | **PC1** | **PC2** |
| F | 91.450 | 21.750 |  | 8.29 | 5.37 |
| p-value | **0.000** | **0.000** |  | **0.001** | **0.007** |
| R-Sq (adj) | 95.01% | 81.37% |  | 60.54% | 47.94% |
|  |  |  |  |  |  |
| Eingenvalue | 4.950 | 2.371 |  | 6.113 | 2.234 |
| Proportion | 0.381 | 0.182 |  | 0.470 | 0.172 |
| Cumulative | 0.381 | 0.563 |  | 0.470 | 0.642 |
|  |  |  |  |  |  |
| SS | 0.045 | -0.415 |  | 0.137 | 0.285 |
| Starch | 0.256 | -0.212 |  | 0.259 | 0.357 |
| Arabinose | 0.185 | 0.061 |  | 0.343 | 0.159 |
| Fucose | -0.128 | -0.416 |  | 0.388 | -0.047 |
| Galactose | -0.317 | -0.245 |  | 0.347 | -0.31 |
| Glucose | 0.142 | -0.07 |  | 0.376 | 0.018 |
| Mannose | -0.38 | 0.168 |  | 0.326 | -0.334 |
| Rhamnose | -0.339 | -0.37 |  | 0.253 | -0.479 |
| Xylose | -0.249 | 0.335 |  | 0.338 | 0.134 |
| Apiose | -0.379 | 0.219 |  | -0.219 | -0.11 |
| Uronics | 0.297 | -0.334 |  | -0.018 | 0.254 |
| RGR-D | -0.336 | -0.275 |  | 0.063 | -0.084 |
| RGR-F | -0.307 | -0.172 |  | -0.219 | -0.474 |

**Supplementary Figure 1**. Typical chromatograms obtained from HPLC-PAD analysis of ethanol soluble sugars (A) and cell wall monosaccharides (B) evaluation. A. Sugars analysis in a Dionex system (ICS 5,000) with a CarboPac PA1 column that was eluted with 150 mM sodium hydroxide and water in a flux of 1 mL/min, which 50% of the elluents were applied in the first 17 min. (sugars separation), after that 100% of NaOH was applied for 5 min. to clean the column and then 5 min. of water to regenerate the column. B. Monosaccharides were separated and identified in a Dionex system (ICS 5,000) with a CarboPac SA10 eluted with 0.8% sodium hydroxide and water in a flux of 1 mL/min. The separation was in the first 12 min., followed by addiction of 200 mM NaOH 75% to wash the column (10 min) and then 150 mM NaOH 0.8% was incorporated to regeneration.

**A**


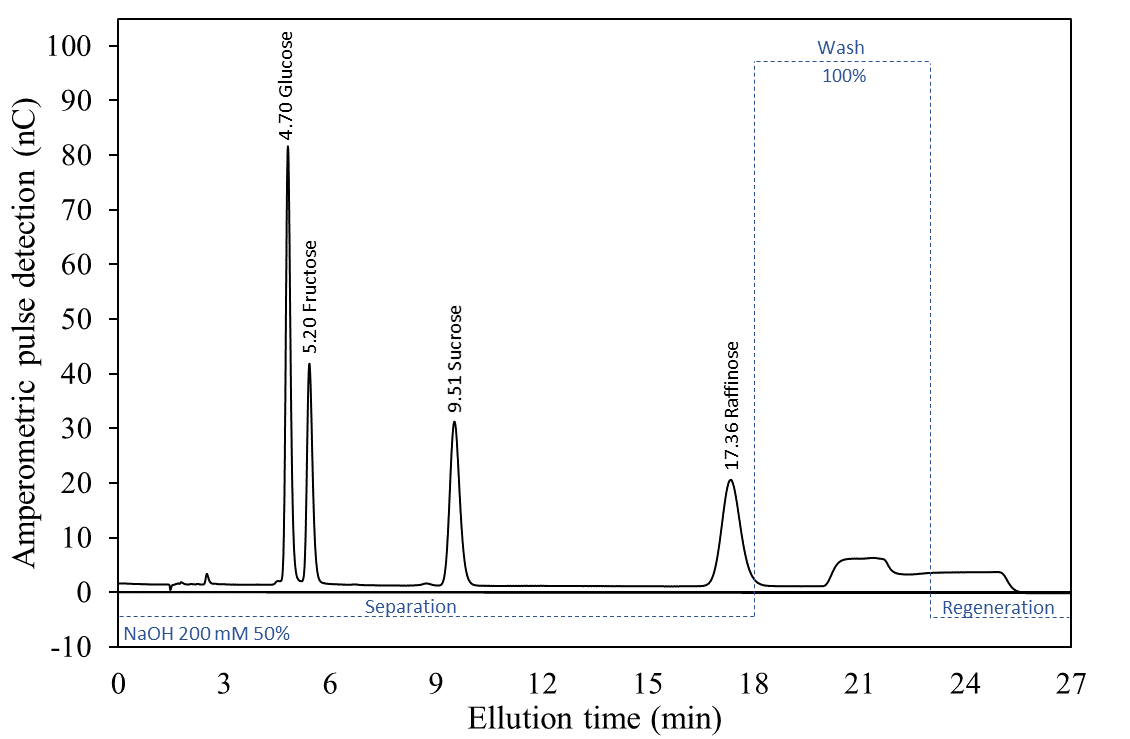


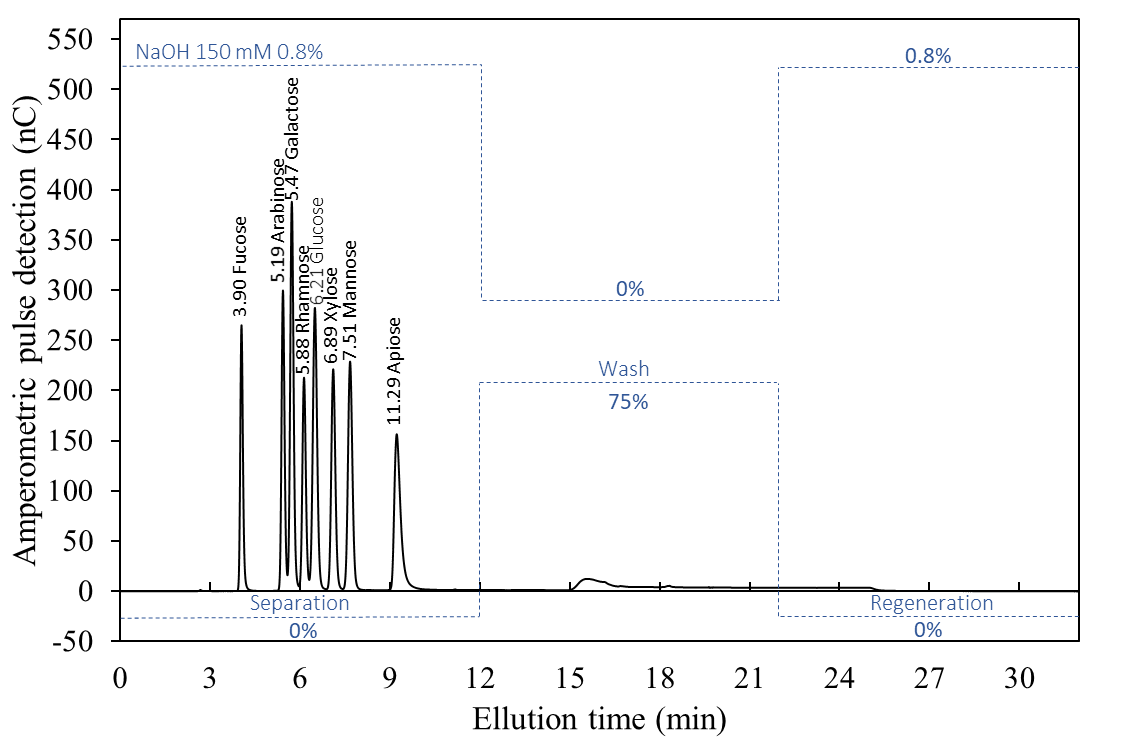


**B**

**Supplementary Figure 2.** Representative chromatograms of duckweed sugar analysis. A. Soluble sugars (Glucose, Fructose, Sucrose and Raffinose) separation in HPLC-PAD in CarboPac PA1 of *L. punctata*. B. Monosaccharides evaluation of *W. borealis* in CarboPacSA10.

**A**


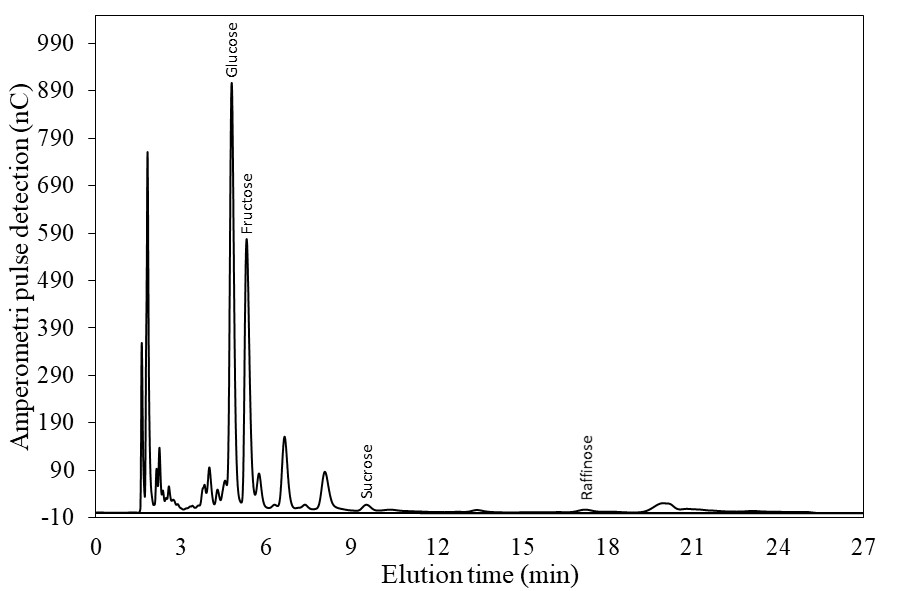


**B**


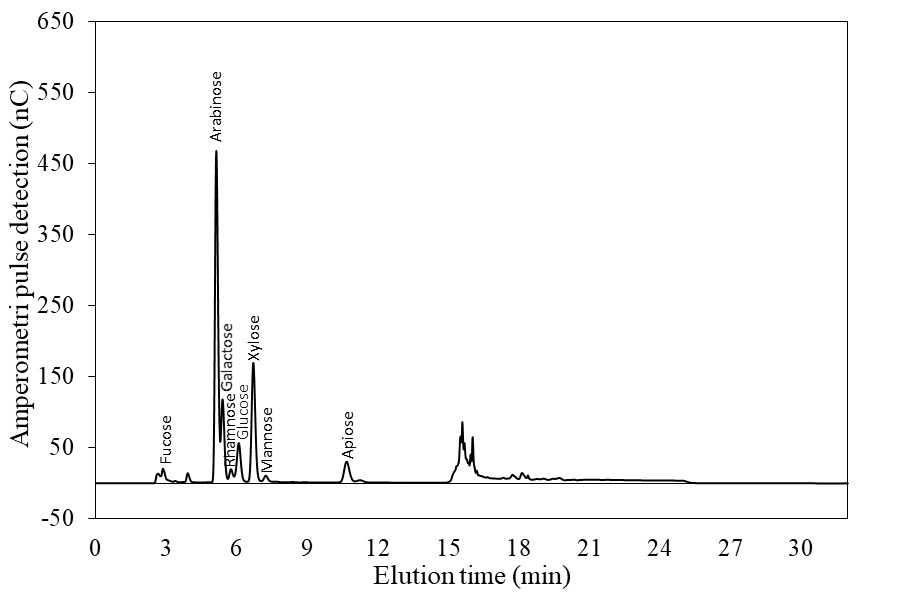


**Supplementary Figure 3.** Metabolic pathways involving the cell wall and non-structural carbohydrates.. The components associated to pectins are pictured in purple and the hemicelluloses in green. The starch synthesis occurs from the ADP-glucose within the chloroplast after the derivation of the carbon assimilated into glucose-1-phosphate. Soluble and several cell wall polymers syntheses depend on the UDP-glucose, formed via fructose-6-phosphate, which is converted to nucleotide sugars (UDP-Arabinose, UDP-Rhamnose, UDP-Glucuronic acid, and UDP-Galactose). The pectins are derived from galacturonic acids having as the main precursor UDP-glucuronic acid and UDP-galactose. As there are different classes of the pectins with different sugars in their composition, the metabolic route of the synthesis of pectins involves the precursors of apiose, arabinose, rhamnose, xylose, and galactose. The hemicellulose is formed from pentoses and hexoses, which are linked to form mannans, xyloglucans, xylans, and arabinans. The mannans and fucose branches are derived directly from the fructose-6-phosphate precursor, whereas the xylans, xyloglucans, and arabinans derive from UDP-glucose. Cellulose is synthesized directly from UDP-glucose. Sucrose is a disaccharide formed by glucose and fructose by the precursors fructose-6-phosphate and UDP-glucose. Raffinose is derived from UDP-galactose.

Source: Adaptations of Alonso et al. (2010), Carpita et al., (2015), Verbančič et al. (2017), and metabolic pathways KEGG 00051 (fructose metabolism and mannose), 00052 (metabolism galactose), 00500 (metabolism starch and sucrose) and 00520 (amino sugar and nucleotide sugar metabolism).

Abbreviations: Fru – fructose, Glc-Glucose, Suc- sucrose, Ara-arabinose, Api- apiose, Fuc- Fucose, Gal – galactose, GlcA – glucuronic acid, Man – mannose, Rha – rhamnose, GalA- galacturonic acid, Xyl – xylose, RG I- rhamnogalacturonan type I, RG-II rhamnogalacturonan type II, XG – xyloglucan, HG – homogalacturonan, AP – apiogalacturonan, and XGA – xylogalacturonan. Black - precursors of sugars; Red - cell wall monosaccharides; Blue - non-structural carbohydrates.


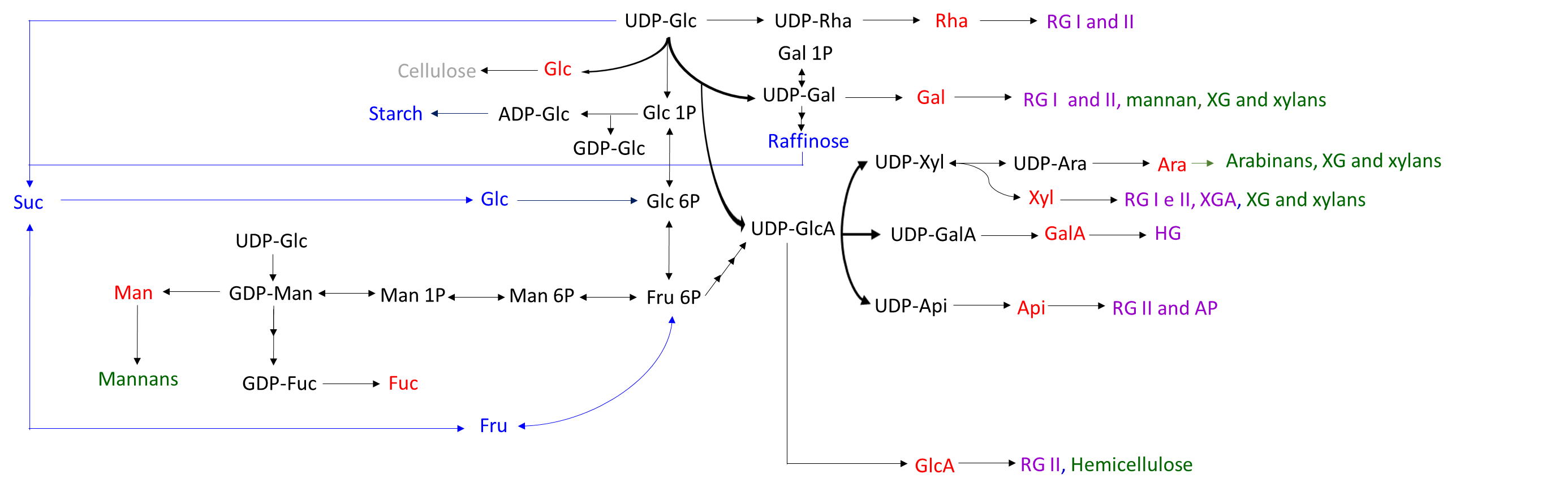

Supplement: Supplementary file 1 [file Table_1.DOCX]
